# Qualitative skeletal correlates of wing shape in extant birds (Aves: Neoaves)

**DOI:** 10.1186/s12862-015-0303-7

**Published:** 2015-02-27

**Authors:** Tobin L Hieronymus

**Affiliations:** Department of Anatomy and Neurobiology, Northeast Ohio Medical University (NEOMED), 4209 St Rt 44, Rootstown, 44272 OH USA

**Keywords:** Aves, Feather, Osteology, Forelimb evolution, Phylogenetic comparative methods, Quill knobs

## Abstract

**Background:**

Among living fliers (birds, bats, and insects), birds display relatively high aspect ratios, a dimensionless shape variable that distinguishes long and narrow vs. short and broad wings. Increasing aspect ratio results in a functional tradeoff between low induced drag (efficient cruise) and increased wing inertia (difficult takeoff). Given the wide scope of its functional effects, the pattern of aspect ratio evolution is an important factor that contributes to the substantial ecological and phylogenetic diversity of living birds. However, because the feathers that define the wingtip (and hence wingspan and aspect ratio) often do not fossilize, resolution in the pattern of avian wing shape evolution is obscured by missing information. Here I use a comparative approach to investigate the relationship between skeletal proxies of flight feather attachment and wing shape.

**Results:**

An accessory lobe of the internal index process of digit II-1, a bony correlate of distal primary attachment, shows weak but statistically significant relationships to aspect ratio and mass independent of other skeletal morphology. The dorsal phalangeal fossae of digit II-1, which house distal primaries VIII and IX, also show a trend of increased prominence with higher aspect ratio. Quill knobs on the ulna are examined concurrently, but do not show consistent signal with respect to wing shape.

**Conclusions:**

Although quill knobs are cited as skeletal correlates of flight performance in birds, their relationship to wing shape is inconsistent among extant taxa, and may reflect diverging selection pressures acting on a conserved architecture. In contrast, correlates of distal primary feather attachment on the major digit show convergent responses to increasing aspect ratio. In light of the diversity of musculoskeletal and integumentary mophology that underlies wing shape in different avian clades, it is unlikely that a single skeletal feature will show consistent predictive power across Neoaves. Confident inference of wing shape in basal ornithurine birds will require multiple lines of evidence, together with an understanding of clade-specific evolutionary trends within the crown.

**Electronic supplementary material:**

The online version of this article (doi:10.1186/s12862-015-0303-7) contains supplementary material, which is available to authorized users.

## Background

**Figure 1:**
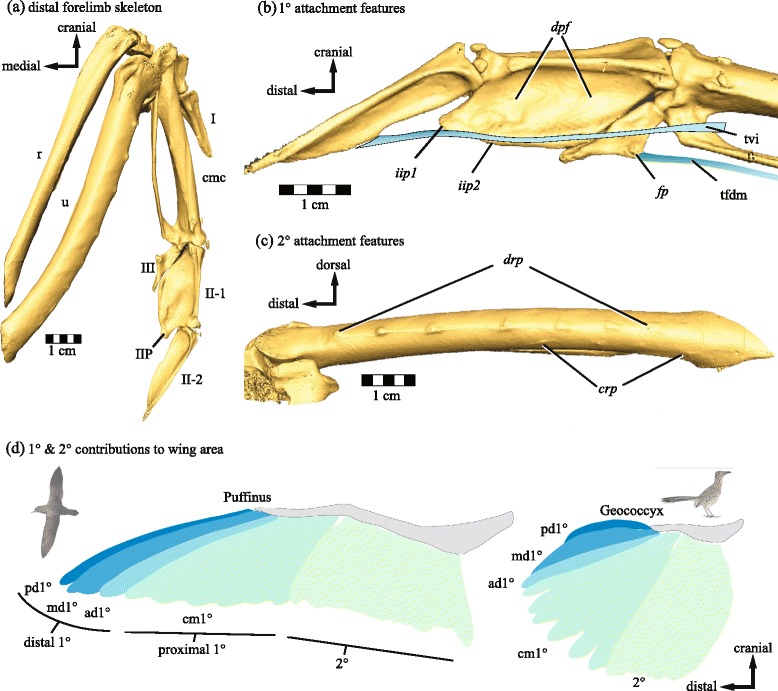
**Bony morphology of feather attachment and composition of wing shape. (a)** – **(c)** Major bony landmarks as visible on a rock pigeon (*Columba livia*). **(a)** Dorsal view of the distal forelimb. I: digit I; II-1: proximal phalanx digit II; II-2: distal phalanx digit II; III: digit III; IIP: internal index process; cmc: carpometacarpus; r: radius; u: ulna. **(b)** 1° attachment features on digits II-III, dorsal view. dpf: dorsal phalangeal fossae (primaries VIII-IX); fp: flexor process of digit III (primaries I-VI); iip1: internal index process (primaries IX-X); iip2: position of caudal lobe of internal index process (primary IX); tvi: tendon of ventral interosseous muscle; tfdm: tendon of flexor digiti minimi muscle. **(c)** 2° attachments on the ulna, caudal view. drp: dorsal remigial papillae; crp: caudal remigial papillae. **(d)** Schematic of flight feather attachment and contribution to wing shape in a pink-footed shearwater *Puffinus creatopus* (left) and a roadrunner *Geococcyx californianus* (right), taxa with roughly equivalent wing area but very different wing shapes and functions. Shading indicates groups of feathers with distinct attachment points. Note that distal primaries form the pointed wingtip in *Puffinus*, while the rounded wingtip in *Geococcyx* is formed by more proximal primaries. pd1°: predigital primary (II-2); md1°: middigital primaries (II-1); ad1°: addigital primary (III); cm1°: carpometacarpal primaries (III + carpometacarpus + tfdm); 2°: secondaries. Bird images: *Puffinus* courtesy M. Taylor (CC-by-SA 3.0) 2009; *Geococcyx* courtesy G. Kramer and USFWS, 2009.

Bird wings represent an extreme modification of the tetrapod forelimb to meet the functional demands of flight. The most apparent change involves the elongation and stiffening of feathers on the forelimb to form an airfoil. The full complement of specially-modified flight feathers contributes more than 85% of total wing area in most extant birds [1], and the overall wing shapes that result from the aggregate of individual flight feather shapes determine critical aspects of aerodynamic function (Figure 1d): Long, narrow wings (high aspect ratio, *AR*) typically lower the drag associated with creating lift, leading to more efficient sustained flight, while increasing wing area for a given body mass (lowering wing loading) leads to more efficient soaring flight at lower speeds [2-4].

Extant birds stand out from the other living flyers (including bats and insects) in their ability to maintain attached, efficient airflow in flapping and soaring flight with high aspect ratio wing shapes and high wing loadings, even at comparatively high Reynolds numbers [1-3,5]. As with many other organismal traits, the evolution of components of flight performance in extant birds, including wing shape, is best understood as gradual change along a continuum or a stepwise accretion of traits [6]. Thus a nuanced understanding of avian wing shape evolution requires information from fossils to constrain evolutionary rates and ancestral character states in deep time [7,8] as a potential explanation for the current ecological and phylogenetic diversity of birds [9].

Efforts to reconstruct evolutionary pattern in wing shape are hindered by the fact that the entire wing is often not preserved as a body fossil, leaving only the pectoral skeletal elements as clues to shape and function in extinct taxa. Inferences of wing shape and flight mechanics in extinct taxa have been primarily based on relationships between whole wing shape parameters and the length of skeletal elements, such as the ratio of humeral and ulnar length [10], or the length of major skeletal segments [11-13]. Cross-sectional properties [14,15] and bone tissue histology [16,17] of both proximal and distal wing elements have also been tested as proxies for wing shape and flight performance.

In contrast, distal limb surface morphology and its relationship to wing shape have a limited history of investigation. Bird wing morphology is unique among the known flying vertebrates (which also include bats and pterosaurs) in that the distal extent of the wing is not demarcated by skeletal features [18,19]. Rather than a membrane that attaches along the length of the pectoral skeletal elements, bird wings are mainly composed of primary (1°) and secondary (2°) flight feathers (remiges, sing. remex), that have restricted, focal attachment points to the underlying skeleton (Figure 1a-c). These attachment points function as mechanical links that transfer aerodynamic forces generated by flight feathers to the limb skeleton [20-24]. Despite their role in the mechanics of flight, remex attachments have not been as thoroughly studied as other potential correlates of flight behavior. Skeletal variability in proximal attachment points (ulnar quill knobs, Figure 1c) has been linked to flightedness [25]. The distal attachments of the 1° feathers, specifically the length of the internal index process (*iip1*, Figure 1b) have been suggested as correlates for aspect ratio [26,27].

The distal portion of a flapping wing produces the greatest aerodynamic force per unit area [28]. The length and shape of distal 1° feathers is thus expected to have a disproportionate effect on force production and induced drag when compared to 2° feathers and proximal 1° feathers. My initial hypotheses are: (a) bony correlates of distal 1° attachment are more prominent in birds with higher *AR*, and (b) bony correlates of proximal 1° and 2° attachment are more prominent in birds with lower *AR*.

The prominence of bony attachment features in general may vary with body size. Mass (*M*), as a proxy for size, is a relevant covariate for any study of skeletal morphology. My initial expectation is that all bony features in the study will be scored as more prominent with increasing mass.

I used phylogenetic distance-based Redundancy Analysis (RDA) [29,30] and variation partitioning (VARPART) [31,32] as means to explore relationships among the several variables simultaneously. RDA works much like a principal components analysis (PCA), which is commonly used to examine several morphological variables at once. RDA has the added benefit of explicitly including information from expected functional relationships. Where PCA reports patterns of greatest morphological variability, RDA reports patterns of greatest morphological variability that are correlated with a second set of variables, in this case *AR* and *M*. VARPART then provides a means of measuring the magnitude of the effect that the second set of variables has on morphology.

After initial exploration of the data, I employed phylogenetic ANOVA [33] to test for consistent relationships between qualitative bony indicators of proximal and distal remex attachment on one hand (Figure 1a-c, Table 1), and aspect ratio and body mass on the other (Figure 1d, Table 1). Phylogenetic ANOVA provides a more conventional and widely used approach to dichotomous hypothesis testing. The results of these tests directly address the question of whether individual categorically-scored bony characters provide a record of *AR* that might be interpreted for extinct taxa.

**Table 1:** **Character scores,** ***AR*** **, and** ***M*** **for taxa used in this study**

**Taxon**	***drp***	***crp***	***dpf***	***iip1***	***iip2***	***fp***	***AR***	***M*** **(kg)**
*Accipiter cooperii*	1	0	1	1	0	1	5.63	0.558
*Accipiter gentilis*	1	0	1	1	0	1	6.23	0.737
*Accipiter nisus*	1	0	1	1	0	1	5.81	0.196
*Accipiter striatus*	1	0	1	1	0	1	5.66	0.139
*Anas crecca*	1	0	0	0	0	1	7.96	0.230
*Anas georgica*	1	0	0	0	0	1	7.20	0.437
*Anas penelope*	1	0	0	0	0	1	8.15	0.770
*Anas platyrhynchos*	1	0	0	0	0	1	7.43	1.09
*Anhinga anhinga*	1	1	0	1	0	1	7.65	0.960
*Anser albifrons*	1	0	0	0	0	1	7.86	2.340
*Anser anser*	1	0	0	0	0	1	7.70	3.650
*Anser indicus*	1	0	0	0	0	1	8.24	2.44
*Ardea cinerea*	2	0	0	1	0	1	7.15	1.21
*Ardea herodias*	2	0	0	1	0	1	8.46	1.65
*Caracara plancus*	2	0	1	1	0	2	6.83	1.30
*Cathartes aura*	2	1	0	1	0	2	7.46	1.64
*Chionis albus*	1	1	1	1	0	2	6.44	0.610
*Ciconia abdimii*	1	0	0	1	0	1	7.00	1.030
*Coccyzus americanus*	2	1	1	1	0	1	6.37	0.059
*Columba palumbus*	1	0	1	1	0	1	7.08	0.495
*Diomedea exulans*	1	1	0	1	1	1	15.0	7.98
*Falco columbarius*	1	0	1	1	0	2	7.01	0.157
*Falco mexicanus*	1	0	1	1	0	2	7.64	0.837
*Falco peregrinus*	1	0	1	1	0	2	8.33	0.798
*Falco rusticolus*	1	0	1	1	0	2	7.88	1.17
*Falco sparverius*	1	0	1	1	0	2	7.70	0.084
*Fregata magnificens*	2	1	2	1	1	2	11.5	1.39
*Fulmarus glacialis*	1	0	1	1	1	1	10.5	0.824
*Gavia immer*	1	1	0	1	0	1	9.97	3.58
*Gavia stellata*	1	1	0	1	0	1	10.3	2.31
*Gyps africanus*	1	1	0	1	1	2	6.88	5.50
*Gyps rueppellii*	1	1	0	1	1	2	7.01	7.30
*Haematopus ostralegus*	1	1	0	1	0	2	8.19	0.460
*Limnodromus griseus*	2	1	1	1	1	1	8.32	0.061
*Megascops asio*	1	0	1	1	0	1	5.87	0.098
*Mergus serrator*	1	1	0	0	0	1	9.64	0.460
*Oceanites oceanicus*	1	1	0	1	0	1	7.27	0.034
*Pachyptila desolata*	1	1	0	1	0	1	8.60	0.155
*Pachyptila turtur*	1	1	0	1	0	1	9.33	0.132
*Passer domesticus*	1	0	0	1	0	1	4.80	0.029
*Pelecanoides georgicus*	1	1	0	1	0	1	7.66	0.122
*Pelecanoides urinatrix*	1	1	0	1	0	1	7.43	0.133
*Pelecanus erythrorhynchos*	2	1	1	1	1	2	9.05	5.09
*Pelecanus occidentalis*	2	1	1	1	1	2	10.8	2.66
*Pelecanus onocrotalus*	2	1	1	1	1	2	8.76	7.30
*Pelecanus rufescens*	2	1	1	1	1	2	7.64	4.80
*Phaethon aethereus*	2	1	1	1	1	1	10.8	0.650
*Phaethon lepturus*	2	1	1	1	1	1	10.0	0.370
*Phaethon rubricauda*	2	1	1	1	1	1	11.2	0.650
*Phalacrocorax aristotelis*	1	1	0	1	0	1	6.90	1.642
*Phalacrocorax atriceps*	1	1	0	1	0	1	6.98	2.23
*Phalacrocorax auritus*	1	1	0	1	0	1	7.58	1.28
*Phalacrocorax carbo*	1	1	0	1	0	1	8.52	2.53
*Phasianus colchicus*	1	0	0	0	0	1	5.21	1.20
*Platalea ajaja*	2	1	0	1	0	1	6.91	1.30
*Plegadis chihi*	2	1	0	1	0	1	6.78	0.418
*Puffinus huttoni*	1	1	0	1	0	1	11.2	0.364
*Puffinus lherminieri*	1	1	0	1	0	1	9.94	0.150
*Puffinus nativitatis*	1	1	0	1	0	1	9.61	0.340
*Puffinus pacificus*	1	1	0	1	0	1	10.2	0.380
*Puffinus tenuirostris*	1	1	0	1	0	1	12.1	0.544
*Rallus longirostris*	1	0	0	0	0	1	5.10	0.220
*Rissa tridactyla*	1	1	2	1	1	1	9.21	0.394
*Stercorarius parasiticus*	1	1	2	1	1	1	9.85	0.419
*Sterna maxima*	2	0	2	1	1	1	12.2	0.256
*Sula dactylatra*	1	0	1	1	1	1	12.2	1.90
*Sula leucogaster*	1	0	1	1	1	1	11.9	0.938
*Sula sula*	1	0	1	1	1	1	11.2	1.10
*Tyto alba*	1	0	1	1	0	1	7.23	0.380
*Uria aalge*	1	0	1	1	0	1	9.34	0.831
*Zenaida macroura*	1	0	1	1	0	1	5.94	0.098
*Ichthyornis dispar*	1	0	1	1	0	1		
*Eocypselus rowei*	0	0	1	1	0	0	(8.15)	
*Parargornis messelensis*	0	0	1	1	0	0	(5.30)	

## Methods

### Remex attachment morphology

Several skeletal features have been implicated in the attachment of flight feathers in past studies [20-24,26]. These features have often been employed in systematics, and as such are known from a broad taxonomic range of living birds. Published binary and multistate categorical scores coded at genus level [34] for six remigial attachment characters (Table 2) were chosen to represent bony features known to be associated with remex attachment. Published scores were then used as a basis to score several additional taxa for a pooled sample of *n* = 71 extant taxa (Additional file 1).

**Table 2:** **Categorically scored bony characters included in this study**

**Character**	**LZ06 #**	**Soft tissue attachment**	**Related remiges**
Dorsal remigial papillae (*drp*)^a-e^	1521	Dorsal 2° remex ligaments	2°
Caudal remigial papillae (*crp*)^a-e^	1522	Ventral secondary remex ligaments, Septum humerocarpale	2°
Dorsal phalangeal fossae (*dpf*)^b,d,f^	1715	Follicles of 1° VIII-IX	1° VIII-IX (md1°)
Internal index process presence/absence (*iip1*)^f^	1721	Interphalango-remigial ligament^h^	1° IX-X (md1° - ad1°)
Internal index process shape (*iip2*)^f^	1722	Interphalango-remigial ligament^h^	1° IX-X (md1° - ad1°)
Flexor process (*fp*)^g^	1731	Tendon of flexor digiti minimi	1° I-VI (cm1°)

Characters related to feather attachment have been identified by several sources (noted below). Corresponding characters described in [34] (LZ06#) included as an aggregate reference.

^a^[20]; ^b^[21]; ^c^[22]; ^d^[23]; ^e^[24]; ^f^[26]; ^g^[35]; ^h^[36].

Published scores for similar characters were also used to score three extinct taxa: *Parargornis messelensis* [37], *Eocypselus rowei* [38], and *Ichthyornis dispar* [39]. Primary feathers are well-preserved for *Parargornis* and *Eocypselus,* which allows estimation of *AR* from the fossils. *Ichthyornis* represents an early occurrence of two feather attachment features (internal index process and dorsal phalangeal fossae, Table 2) that are homologous with extant examples.

Taxon scores for attachment morphology were submitted to principal coordinates analysis (PCO) using a Hamming distance metric with Cailliez correction for negative eigenvalues [29]. This analysis and all subsequent analyses were performed using R 2.15.2 [40]. PCO provides a semi-quantitative view of a set of categorical characters that is amenable to linear modeling [29]. Correlations between the original character scores and PCO scores provides a projection of the original categorical variables on the PCO axes [41] analogous to loadings in principal component analysis (PCA). The PCO scores for each taxon form the response matrix **Y**. Extinct taxa were included in this portion of the analysis.

### Wing shape and body mass

Measurements of mass (*M*), wing span (*b*), and wing area (*S*) for the study taxa were taken from published sources [1,42,43]. Span and area were recombined into aspect ratio (*AR*, = *b*^2^/*S*). *AR* and *M* were log_e_ transformed to achieve normality (tested with Shapiro-Wilk W, *p*_normal_ = 0.64 & 0.61, respectively). These two variables form the explanatory matrix **X**. Estimates of *AR* from *Parargornis* and *Eocypselus* were excluded from this matrix and from subsequent steps.

### Phylogenetic context

This study accounted for phylogenetic error covariance using a phylogenetic generalized least squares (PGLS) approach [44]. A subset of 1,000 trees containing the study taxa was obtained from a published avian supertree set [9]. A separate phylogenetic covariance matrix was derived from the topology and branch lengths of each tree [45]. Phylogenetic signal (λ_*i*_) for each tree was jointly estimated for all variables [46]. Estimated values of λ_*i*_ were tested against λ_*i*_ = 0 and λ_*i*_ = 1 by a likelihood-ratio chi-squared test with one degree of freedom. Estimated values of λ_*i*_ were used to scale off-diagonal branch lengths in phylogenetic covariance matrices.

All variables were phylogenetically centered around an estimate of ancestral character state [47] for each tree *i*. Because *AR* and *M* have heterogeneous units, they were ranged to (−1, 1) after centering. Centered and ranged variables were then PGLS transformed using the inverse square root of each phylogenetic covariance matrix (**C**_i_^**-1/2**^). Because the explanatory and response matrices **X** and **Y** are multiplied in the course of VARPART and RDA analyses, this transformation is equivalent to multiplication by **C**^−1^ in standard PGLS approaches. The transformed variables can be analyzed by standard parametric linear approaches while accounting for expected phylogenetic error covariance among related species.

### Statistical analysis

Relationships between bone morphology and wing shape were initially evaluated using VARPART [31,32,48] and RDA [29,48]. In this study, VARPART partitions the variation in bony morphology into components that are ‘explained’ by correlation with *AR* and *M*, and a residual component that is not correlated to either. RDA then provides a PCA-like view of the components of bony morphological variation that are correlated to *AR* and *M* (the canonical axes) and the residual components (the non-canonical axes).

VARPART and RDA were run with an iteration to incorporate each of the *i* trees in the sample, to accommodate uncertainty in phylogeny. Using the set of 1,000 trees provides a distribution of adjusted R^2^ scores and axis loadings. Comparison within this distribution serves as a sensitivity analysis of VARPART and RDA to assumptions of tree topology and branch length. As the ordination results are not nested, results for individual trees are not directly comparable. VARPART and RDA results for a single tree with adjusted R^2^ values closest to the means for all terms was taken as a representative ordination.

Eigenvectors from RDA were used as a basis to plot taxon scores in constrained reduced space [15,30] on the canonical axes. In a sense, this step takes taxon scores from an unconstrained ordination that reflects patterns of shared variability in bony morphology (PCO), and projects those scores onto a new set of axes that reflect patterns of shared variability in bony morphology that are correlated with *AR* and *M* (RDA). The constrained reduced space provides a concise summary of variation in bone morphology that is directly tied to variation in wing shape. Extinct taxa were placed on this ordination space using their PCO scores.

One-to-one relationships between skeletal features and *AR* or *M* identified from the ordination were further tested in phylogenetic context using phylogenetic ANOVA [33,46].

## Results

### Representation of categorically scored bony morphology by Principal Coordinates

**Figure 2:**
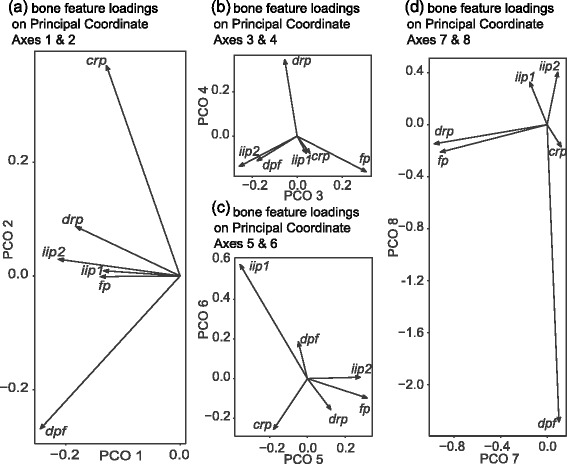
**Biplots of bone morphology characters on successive PCO axes.** Insets show bone feature loadings on Principal Coordinate Axes **(a)** 1-2, **(b)** 3-4, **(c)** 5-6, and **(d)** 7-8. *drp*: dorsal remigial papillae; *crp*: caudal remigial papillae; *dpf*: dorsal phalangeal fossae; *iip1*: presence/absence of internal index process; *iip2*: shape of internal index process; *fp*: flexor process digit III.

The first twelve PCO axes are used to represent bony morphology for subsequent analyses. Of these axes, the first eight each represent greater than 1% of total variability (Figure 2). Eigenvalues for PCO axes are presented in Table 3. Relationships between the unconstrained PCO space and the original binary or categorically scored bone morphology characters are presented in Table 4.

**Table 3:** **Eigenvalues of PCO axes used to represent categorically scored bony characters**

	**Relative eigenvalue**	**Cumulative eigenvalue**
PCO1	0.19	0.19
PCO2	0.14	0.33
PCO3	0.07	0.40
PCO4	0.06	0.45
PCO5	0.05	0.50
PCO6	0.02	0.52
PCO7	0.02	0.54
PCO8	0.02	0.56

**Table 4:** **Projection of the original morphological characters onto the first eight PCO axes**

	**PCO1**	**PCO2**	**PCO3**	**PCO4**	**PCO5**	**PCO6**	**PCO7**	**PCO8**
*drp*	−0.58	0.25	−0.16	0.98	0.29	−0.26	−1.54	−0.13
*crp*	−0.41	1.05	0.17	−0.22	−0.42	−0.43	0.19	−0.15
*dpf*	−0.78	−0.77	−0.52	−0.31	−0.11	0.31	0.16	−2.01
*iip1*	−0.43	0.03	0.12	−0.22	−0.83	0.96	−0.24	0.29
*iip2*	−0.68	0.08	−0.75	−0.39	0.65	0.01	0.15	0.35
*fp*	−0.45	0.00	0.90	−0.45	0.73	−0.16	−1.46	−0.19

This matrix was used to transform the loadings of PCO axes in the RDA ordination into a biplot of the original morphological variables (Figure 2a).

### Phylogenetic signal

**Figure 3:**
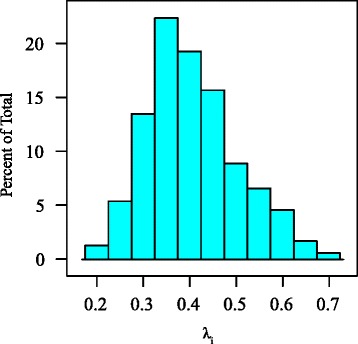
**Histogram showing distribution of joint** **λ**_i_ **values estimated for the tree sample.**

**Figure 4:**
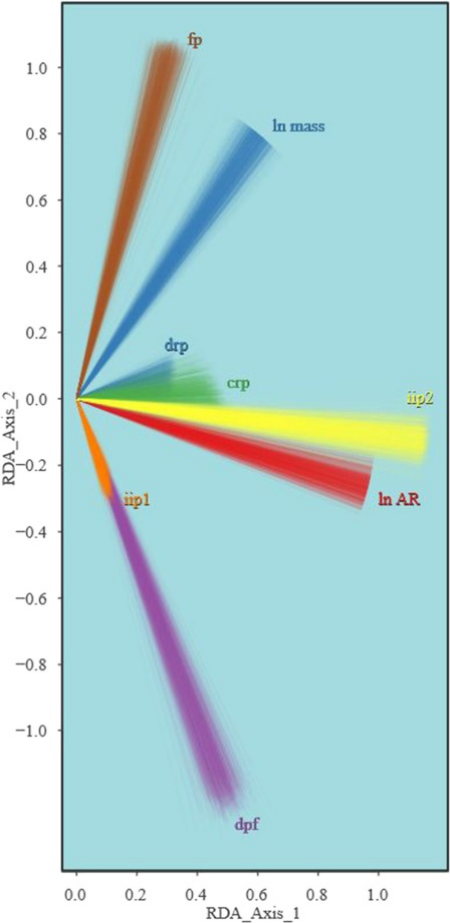
**Biplots showing the distribution of bone and wing shape character loadings for the tree sample.** *drp*: dorsal remigial papillae; *crp*: caudal remigial papillae; *dpf*: dorsal phalangeal fossae; *iip1*: presence/absence of internal index process; *iip2*: shape of internal index process; *fp*: flexor process digit III; *AR*: aspect ratio; *M*: mass.

Joint estimates of λ_*i*_ for the study data are significantly different from both zero and one (*p <* 0.001) for all phylogenetic trees considered in the study, and are normally distributed around a mean λ_*i*_ = 0.67 (Figure 3). Variable loadings in the redundancy analysis (RDA) ordination space for all trees are clustered around similar values (Figure 4).

### Aspect ratio is linked to variability in skeletal features of the manus

**Figure 5:**
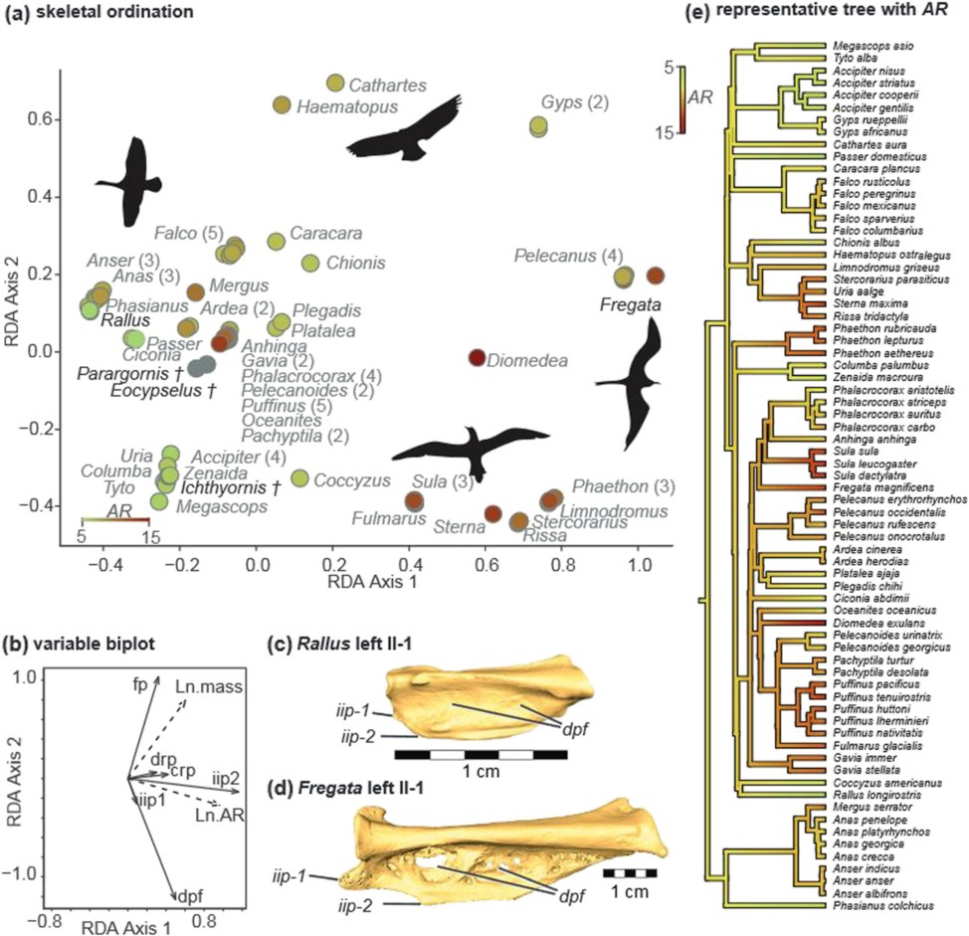
**Summary of VARPART and RDA results. (a)** Plot of taxon RDA scores. Hue and shading of points denotes aspect ratio. Silhouettes show representative wing shapes for *Anser*, *Cathartes*, *Sula*, and *Fregata*. **(b)** Inset biplot representing PGLS correlations among response matrix **Y** (solid rays) and explanatory matrix **X** (dashed rays) in the canonical RDA axes. Note the strong positive correlation between aspect ratio (*AR)* and distal primary attachment features (*iip2). drp*: dorsal remigial papillae; *crp*: caudal remigial papillae; *dpf*: dorsal phalangeal fossae; *iip1*: presence/absence of internal index process; *iip2*: shape of internal index process; *fp*: flexor process digit III. **(c)** – **(d)** Proximal phalanges of digit II (II-1) from *Rallus* and *Fregata* in dorsal view, show morphological disparity at the extremes of the first RDA axis. **(e)** Representative tree topology with an ancestral character state reconstruction of *AR*—hues and shading correspond to **(a)**.

VARPART and RDA identified significant components of variation in bony feather attachment morphology that are best explained by *AR* (R^2^ = 0.065, Table 5). Although the coefficient of determination is low, this reflects the relationship of *AR* to all of the included skeletal features simultaneously. Permutation tests identified both the first canonical axis (Figure 5a,b) and the loadings of *AR* and *M* along the first axis as significantly different from random (*p* ≤ 0.05, Table 6). These results, along with the loadings shown in Figure 5b, show that although the statistical model has poor predictive power, there is an association between *AR* and more prominent bony feather attachment features in the hand. This association is convergent across multiple neoavian lineages (Figure 5a,d).

**Table 5:** **VARPART results for single representative tree, with the range observed in the tree sample**

**Model**	**Adjusted R** ^2^	**Upper adj. R** ^2^	**Lower adj. R** ^2^
*AR*	0.065**	0.057	0.073
*M*	0.033*	0.022	0.041
*AR + M*	0.082	0.070	0.092
*AR | M*	0.050	0.041	0.057
*M | AR*	0.016	0.011	0.020
*AR ∩ M*	0.017	0.011	0.023
Residuals	0.918	0.908	0.930

**Significant by permutation test at *p* ≤ 0.01.

*Significant by permutation test at *p* ≤ 0.05.

**Table 6:** **RDA axis eigenvalues for single representative tree**

**Axis**	**Eigenvalue**	**Relative eigenvalue**
1^st^ canonical axis	4.46 × 10^−4^ **	8.3%
2^nd^ canonical axis	1.39 × 10^−4^	2.6%
1^st^ non-canonical axis	1.32 × 10^−3^	25%
2^nd^ non-canonical axis	1.28 × 10^−3^	24%
3^rd^ non-canonical axis	5.86 × 10^−4^	11%
4^th^ non-canonical axis	4.68 × 10^−4^	8.7%
5^th^ non-canonical axis	4.01 × 10^−4^	7.5%
6^th^ non-canonical axis	1.86 × 10^−4^	3.5%
7^th^ non-canonical axis	1.66 × 10^−4^	3.1%
8^th^ non-canonical axis	1.16 × 10^−4^	2.2%
9^th^ non-canonical axis	1.08 × 10^−4^	2.0%

**Significant by permutation test at *p* ≤ 0.01.

Although *M* has a non-random effect on the ordination of bony morphology (Table 5), mass primarily loads along the second canonical axis (Figure 5b), which is only marginally distinct from a random rearrangement of the original PCO axes (*p* = 0.07).

### Shape of the caudal margin of digit II is a correlate of aspect ratio and mass

The presence of a caudal lobe of the internal index process (*iip2*, Figure 1b) is associated with significantly different *AR* and *M* (*p* = 0.001 & *p =* 0.013, respectively; phylogenetic ANOVA using the representative tree with 1,000 simulations and Holm multiple comparisons adjustment). As scored for this analysis, presence or absence of the flexor process of digit III (*fp*) and the prominence of dorsal phalangeal fossae (*dpf*) do not show significant relationships to *AR* (*p* = 0.8 & *p* = 0.4, respectively) or *M* (*p* = 0.07 & *p* = 0.8).

### Modeled *AR* for extinct taxa are inconclusive

Fitted *AR* for *Eocypselus*, *Parargornis* (7.3), and *Ichythyornis* (7.8) were all close to the value inferred for the root of the neoavian tree in the representative ordination (7.6, Figure 5d). These values represent an underestimation for *Eocypselus* (inferred *AR* of 8.2 from feather impressions) [38], and an overestimation for *Parargornis* (inferred *AR* of 5.3) [37]. The weak fit of *AR* to the full RDA model, which contains all of the morphological features, does not allow any conclusions to be drawn regarding wing shape in *Ichthyornis*.

## Discussion

### Variation in feather attachment morphology

Loadings of the PCO axes identify prominent patterns of variation in bony flight feather attachment features in living birds (Figure 2a-d). The first PCO axis captures a general trend of linked presence or absence among most of the bony characters. The second PCO axis shows a pattern of divergence in which several taxa possess caudal remigial papillae (*crp*) but not dorsal phalangeal fossae (*dpf*), and vice versa, which points to a trade-off between the prominence of 2° and distal 1° feather attachments, respectively. The third PCO axis captures a similar tradeoff between distal (*iip2*, *dpf*) and proximal (*fp*) 1° feather attachments. The fourth PCO axis accounts for variability in dorsal remigial papillae (*drp*) that does not co-occur with any of the above patterns. The remaining axes account for residual variation that occurs as exceptions to the abovementioned trends.

### Phalangeal morphology and distal primary attachment

The simple presence/absence character score for *iip1* (Figure 1b) used in this study obscures a broad taxonomic range of shape and relative size in this feature. The ‘caudal lobe’ (*iip2*) [34] is only found in taxa with very prominent internal index processes (IIP), and thus may also serve as a crude proxy for the length of the IIP, as defined in the strict sense. Because the IIP and its caudal lobe are attachment sites for ligaments that hold primaries VIII, IX and X to the major digit, the significant relationship between these features and *AR* suggests a relatively straightforward interpretation: longer distal 1° feathers contribute to increased span, drive up *AR* (Figure 1d), and presumably exert greater stress on their attachment sites, resulting in more prominent bony features (Figure 5d).

The relationship between IIP size and *AR* has been suggested to follow from a hypothesized role of the IIP as a guide for the tendon of the ventral interosseus muscle (tvi, Figure 1b) [26]. By this interpretation, increased IIP length would result in increased moment arm for adduction of the distal phalanx and its attached predigital 1° feather. However, the potential performance benefits of increased moment arm for predigital 1° adduction in flapping flight are unclear, and the role of the IIP as a remigial ligament attachment site, although supported by anatomical data, has not previously been considered. Further anatomical and histological studies will undoubtedly shed more light on the functional significance of the IIP.

### Ulnar remigial papillae

**Figure 6:**
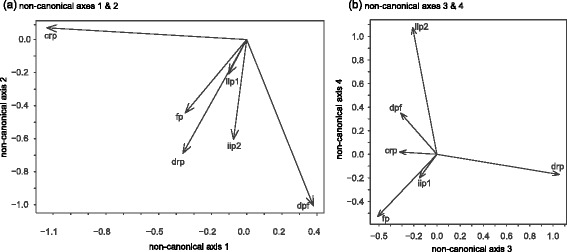
**Non-canonical axes from RDA, representing residual variation in bony morphology that is not related to** ***AR*** **or** ***M*** **.** Plots show non-canonical axes **(a)** 1-2, and **(b)** 3-4.

**Figure 7:**
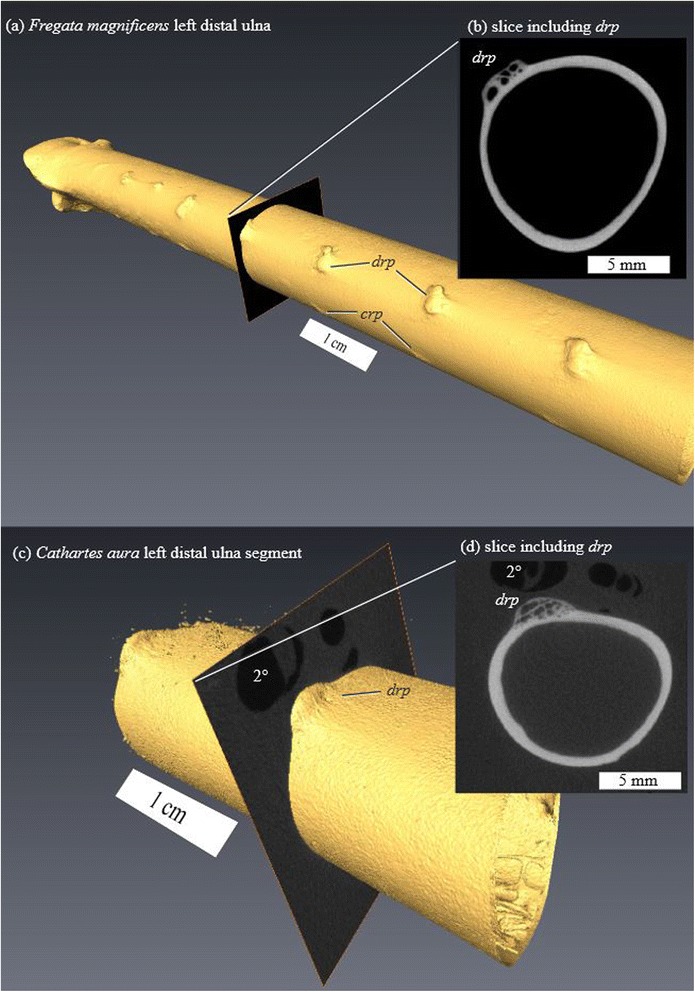
**MicroCT surfaces and slices through dorsal remigial papillae (** ***drp*** **). (a-b)** *Fregata* (CM S13606) and **(c-d)** *Cathartes*, showing open spaces isolated from the main medullary cavity of the ulna. *Cathartes* specimen is embedded in plastic for histological sectioning—feather pulp cavities appear as void spaces in the slice. *crp*, caudal remigial papillae; 2°, pulp cavity of secondary remex.

The prominence of ulnar remigial papillae does not show a clear relationship to *AR* or *M* (Figure 5b). Biplots of the original morphological variables on the non-canonical (residual) axes from RDA (Figure 6) show that *crp*, *dpf*, and *drp* show independent patterns of variation on the first, second, and third non-canonical axes, respectively. Distal limb pneumatization (‘hyperpneumatization’ [49]) is a possible confounding variable for these features, as many of the hyperpneumatized taxa in the study also show prominent *drp* and *dpf*. In many birds with distal limb pneumaticity, subcutaneous airsacs lie adjacent to the follicles of 1° and 2° remiges at their attachments [49]. Cross-sections of *drp* in these taxa reveal substantial erosion rooms within the papillae themselves that are independent of the medullary cavity of the ulna (Figure 7). In a similar manner, *dpf* of hyperpneumatized taxa (Figure 5d) show prominent excavations associated with air sacs surrounding follicles of the distal 1° remiges.

A single interpretation of quill knobs cannot be extended to address all taxa included in the present study. In some taxa (e.g. *Ardea*), prominent *drp* are not associated with pneumatization of the distal forelimb—the same is also true of the *dpf* of *Rissa*, *Stercorarius*, and *Sterna* [49]. Although the morphological pattern of quill knobs scored in this study suggests that the overlying system of feather attachment ligaments is homologous among extant birds, the patterns of bone growth and resorption that produce different bony character states are clearly homoplastic. Quill knobs may appear similar in these independent occurrences, but their presence may very well serve different purposes in different clades.

### Wing shape and many-to-one mapping

The coefficient of determination for *AR* found by VARPART (Table 5) is noteworthy despite its low value for two reasons. First, the preservation of any signal that can be detected through the noise and low resolution of several anatomically independent, categorically scored characters across the diversity of neoavian birds is encouraging. The relationships between phalangeal morphology and *AR* detected by RDA warrant further investigation with more precise measures of morphology.

Second, although the relationship between discrete bony attachment characters and *AR* is weak, it is not a great deal weaker than previous models that employed brachial index (BI, humerus length/ulna length) as a predictor of *AR* when phylogeny was taken into account [50]. Difficulty in predicting wing shape from skeletal features in general may be a reflection of distinct functional relationships within different neoavian clades. If this is the case, predictive power may only be found by considering more phylogenetically restricted samples.

Extant birds may also display a pattern of many-to-one mapping [51], in which several distinct combinations of element and feather lengths may result in functionally equivalent wings with similar *AR*. Wing span, as a component of *AR*, is largely a summation of the lengths of humerus, ulna, manus, and distal 1° feathers (Figure 1d). The low predictive power of both the current RDA model and BI may reflect the fact that each model excludes important skeletal information from either the proximal or distal segments of the wing. The consilience of results from this study and previous work points in two directions for future studies: look for relationships in more restricted regions of the avian tree, and begin with skeletal data that address all four of the variable-length segments in the avian wing.

## Conclusions

Although the ulnar papillae have been the most frequently cited correlates of feather attachment, their link to overall wing shape is problematic, and likely driven by divergent trends in different clades of neoavian birds. Homoplasy of prominent ulnar papillae may be better thought of as a reflection of conserved ligament architecture, rather than a convergent flight-related functional signal.

The internal index process of digit II-1 and associated attachment structures have previously been overlooked as correlates of flight feather attachment, but show a clear relationship to aspect ratio and the shape of the distal primaries. This relationship is apparent even at the low level of precision available in binary and categorical character scores. A more fine-grained approach to quantifying the size and shape of distal primary attachment features would provide a further test of the sensitivity of phalangeal morphology as a correlate of wing shape.

Feather attachment features provide a novel line of evidence for wing shape and function, alongside previously established relationships such as element length indices [10-13], cross-sectional properties [14,15], and bone tissue organization [16]. Combining evidence from feather attachment morphology with other forms of skeletal evidence, and restricting the phylogenetic scope of modeling efforts, may yet yield accurate skeletal predictors of wing shape to shed light on the evolution of flight mechanics within crown neoavian birds as a dynamic, divergent process [52].

## Availability of supporting data

The data sets and R language scripts supporting the results of this article are available in Dryad (http://dx.doi.org/10.5061/dryad.2p31f).

## Additional file

Additional file 1:
***Hieronymus flight feather Table S7.xls*** **: Excel spreadsheet of taxa included in the study.** Coding abbreviations: LZE—Livezey & Zusi (2007) exemplar taxon; LZC—Livezey & Zusi (2007) exemplar congener; OST—coded from osteological specimen. Institutional abbreviations: CM—Carnegie Museum of Natural History. Wing data reference abbreviations: W’77—Warheit (1977); HB’99—Hertel & Ballance (1999); P’01—Pennycuick (2001); P’08—Pennycuick (2008).

## Abbreviations

1: Primary feather
2: Secondary feather
ad1: Addigital primary feather
cm1: Carpometacarpal primary feathers
cmc: carpometacarpus
*crp*: Caudal remigial papillae
*dpf*: Dorsal phalangeal fossae
*drp*: Dorsal remigial papillae
*fp*: Flexor process digit III
I: Manus digit I
II-1: Proximal phalanx of manus digit II
II-2: Distal phalanx of manus digit II
III: Manus digit III
IIP: Internal index process (feature)
*iip1*: Internal index process (character score)
*iip2*: Caudal lobe of internal index process (character score)
*M*: Body mass
md1: Middigital primary feathers
PCO: Principal coordinates analysis
pd1: Predigital primary feather
PGLS: Phylogenetic generalized least-squares
r: Radius
*AR*: Aspect ratio
RDA: Redundancy analysis
tfdm: Tendon of flexor digiti minimi muscle
tvi: Tendon of ventral interosseous muscle
u: Ulna
VARPART: Variation partitioning
